# Cemented all-poly tibia in resource constrained country, affordable and cost-effective care. Is it applicable at this era? Review article

**DOI:** 10.1016/j.amsu.2019.09.010

**Published:** 2019-09-27

**Authors:** Vickash Kumar, Obada Hasan, Masood Umer, Naveed Baloch

**Affiliations:** a Fellow of Arthroplasty Society(PAS), South City Hospital Karachi, Pakistan; b Department of Surgery, Section of Orthopedics, The Aga Khan University Hospital, Pakistan

**Keywords:** All-poly tibia, TKR, TKA, Review

## Abstract

Osteoarthritis of knee is a progressive disease requiring total knee replacement in advanced stage. TKR is being performed in high numbers in developing countries as well. It carries significant economic burden on health system including high cost of implants. Initially, tibial components were cemented all polyethylene monoblock constructs. Subsequent studies showed excellent long term follow up in terms of durability up to 20 years.Successive studies reported aseptic loosening as the cause of failure but such studies failed to address factors responsible for failure other than implant. Cemented metal-backed non-modular tibial components (MBT) are implants in current use. They provide modularity in terms of polyethylene thickness, stems wedges. A literature reported cost saving of $1.17 million, by operating 16,500 total joints using all poly-tibial tibial component rather than metal backed tibial component. studies have reported no significant difference in terms of survivorship, function and backside wear.

**Methods:**

For this study only English written articles were included. Studies included case reports, case series, RCTs and systemic reviews related to all polyethylene tibial components. Articles reporting all levels of evidence – Level I to IV- were included as part of our research. PubMed, Google Scholar and Cochrane Reviews databases from 2000 to 2016 were searched for studies.

**Results:**

Information was gathered and thoroughly studied from 30 articles with overall result in favor of the APTC implant.

**Conclusion:**

All polyethylene tibial component (APTC) is an appealing and cost effective alternative, and is associated with the excellent survivorship and lower risk of revision. In light of the present-day economic evidence and long-term functional outcome, all-polyethylene should be in more use than metal backed especially in resource-constrained setting.

## Highlights

• Total Knee Arthroplasty revolutionized the management of end-stage arthritis of the knee.
• Tibial components were cemented all polyethylene monoblock with decreased bone resection.
• Cemented metal-backed non-modular tibial components (MBT) were subsequently introduced.
• Incremental cost can be a devastating problem for the patients at resource-constrained settings.
• In this era of competitive implant's industries, cost-effective options should be explored and adapted.

## 1 Historical background: one of the leading causes of global disability

Osteoarthritis (OA) is one of the leading causes of global disability and one of the most common degenerative conditions affecting knee joint, limiting its motion and necessitating surgical intervention [1,2]. A recent study showed marked improvements in pain and functional disability with surgical management when compared with non-surgical management at 12 months [3].

Dutch Institute for Public Health (RIVM) has mentioned the incidence rate of 1.18 and 2.8 per 1000/year. COPCORD Studies conducted in Pakistan, India and Bangladesh showed increased prevalence of OA Knee among urban population then rural population [4].

Osteoarthritis, as a progressive disease requiring intervention, pose economic burden on health system. More than 640,000 procedures performed annually, costing about $10.2bn (£8.3bn, €9.6bn) [5]. In last 2 decades there was tremendous increase of 161% in number of total knee arthroplasties (TKA) in UK alone from 93,230 to 243,802 procedures annually [6].

**Fig. 1:**
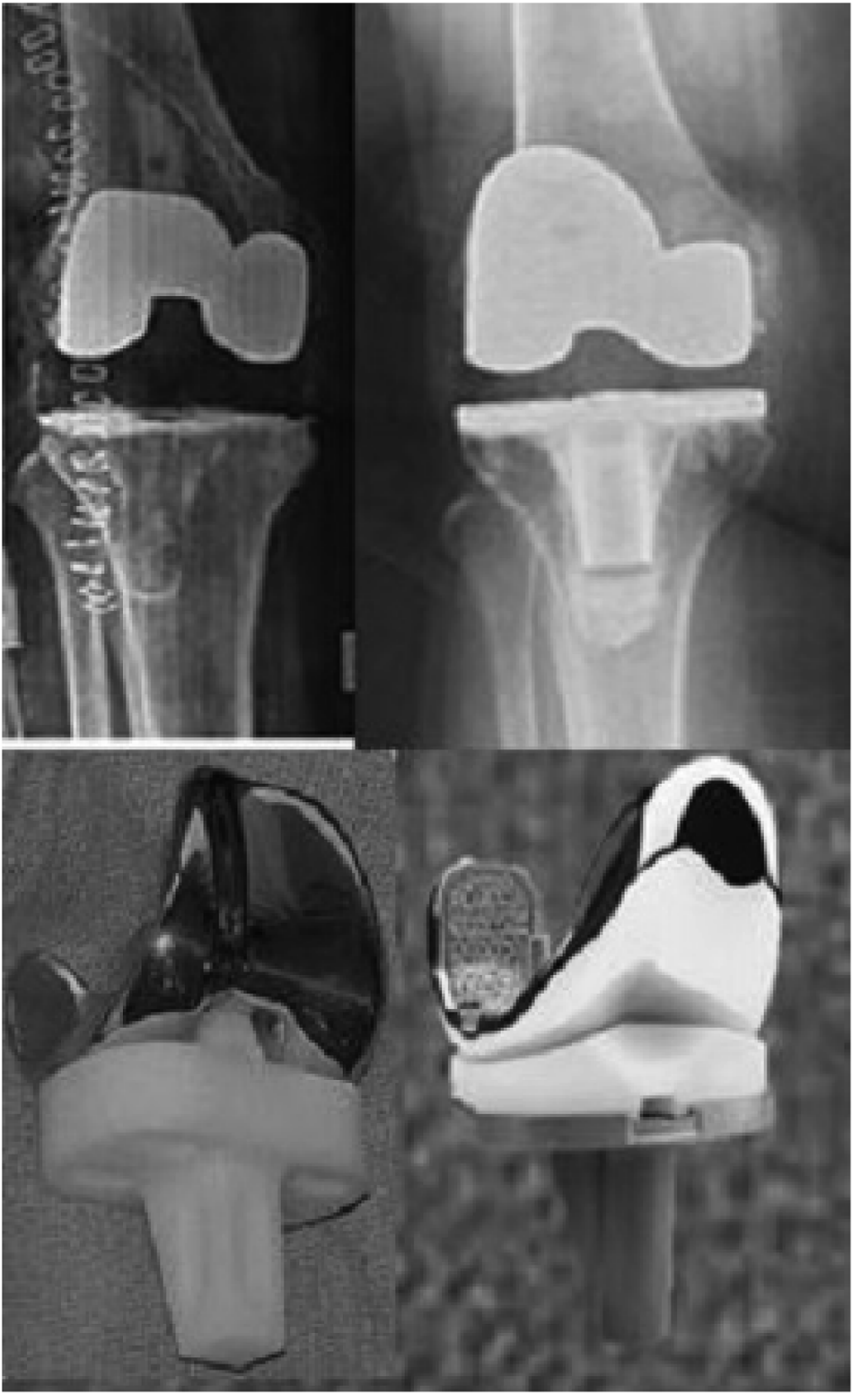
Comparison of both implants as well as their appearance on plain radiographs. All-poly tibia is radiolucent (the left part of the figure) while both have metal backed femoral implant.

Originally, tibial components were cemented all polyethylene monoblock (APT) constructs i.e. thicker polyethylene with decreased bone resection in 1960s and it showed excellent survival rates (Fig. 1) [7]. Main cause of failure and revision was aseptic loosening of the tibial component, which is same reason of failure of other implants as well [[7], [8], [9],^58^]. Cemented metal-backed non-modular tibial components (MBT) were subsequently introduced in the mid-80s providing intraoperative versatility in terms of polyethylene thicknesses, and addition of stems and wedges but increase cost [[8], [9], [10]].

National Joint Registry [11] of England and Wales (2004) mentioned limited use of all polyethylene tibial component (APTC) in 0.6% (248) of the 42,791 recorded cases. Health East Joint Registry, documented APTC usage ranging between 3.9% and 12.9% annually and was 10.7% in 2008 [12,13]. Table 1 summarizing some of the studies done about this design.

**Table 1:** Studies describing the long term results of All-Poly Tibia design.

Author/year [Ref.]	Study design	Sample size (patients)	Follow up (years)	Results
Bruni et al. 2016 [50]	Retrospective	273	10	87% survivorship
Gustke 2017 [51]	Retrospective	227	5.6	100% (no loosening)
Yassin et al., 2015 [52]	Retrospective	22	10	92% survivorship
Gudnason et al., 2014 [29]	SKAR^a^	11,722	10	bAPC > MBTC
Murray et al., 2014 [53]	RCT	207	10	APC < MBTC
Kremers et al., 2014 [54]	Prospective	11,584	20	APC > MBTC
Gioe 2007 [26]	RCT	97	10	91.6% survivorship
Gioe et al., 2007 [55]	prospective	443	14	99.4% survivorship

a Swedish Knee Arthroplasty Register.

b All-Poly Tibia Component, Metal Backed Tibial Component.

## 2 Is there any place of the (APTC) in this era?

### 2.1 Why it failed initially?

Polyethylene was only 5.0–7.5 mm thick initially; the relative deficiency of joint congruency and the inadequate surface coverage of tibia were main reasons for its failure mentioned in early studies. Inadequate soft-tissue balancing, lack of proper procedure, including component mal-alignment and improper fixation was also reasons for early failure [[14], [15], [16], [17], [18], [19]]. Needless to say, correct sizing is crucial step determining overall alignment and survival of the implant [20].

### 2.2 Economic analysis

**Fig. 2:**
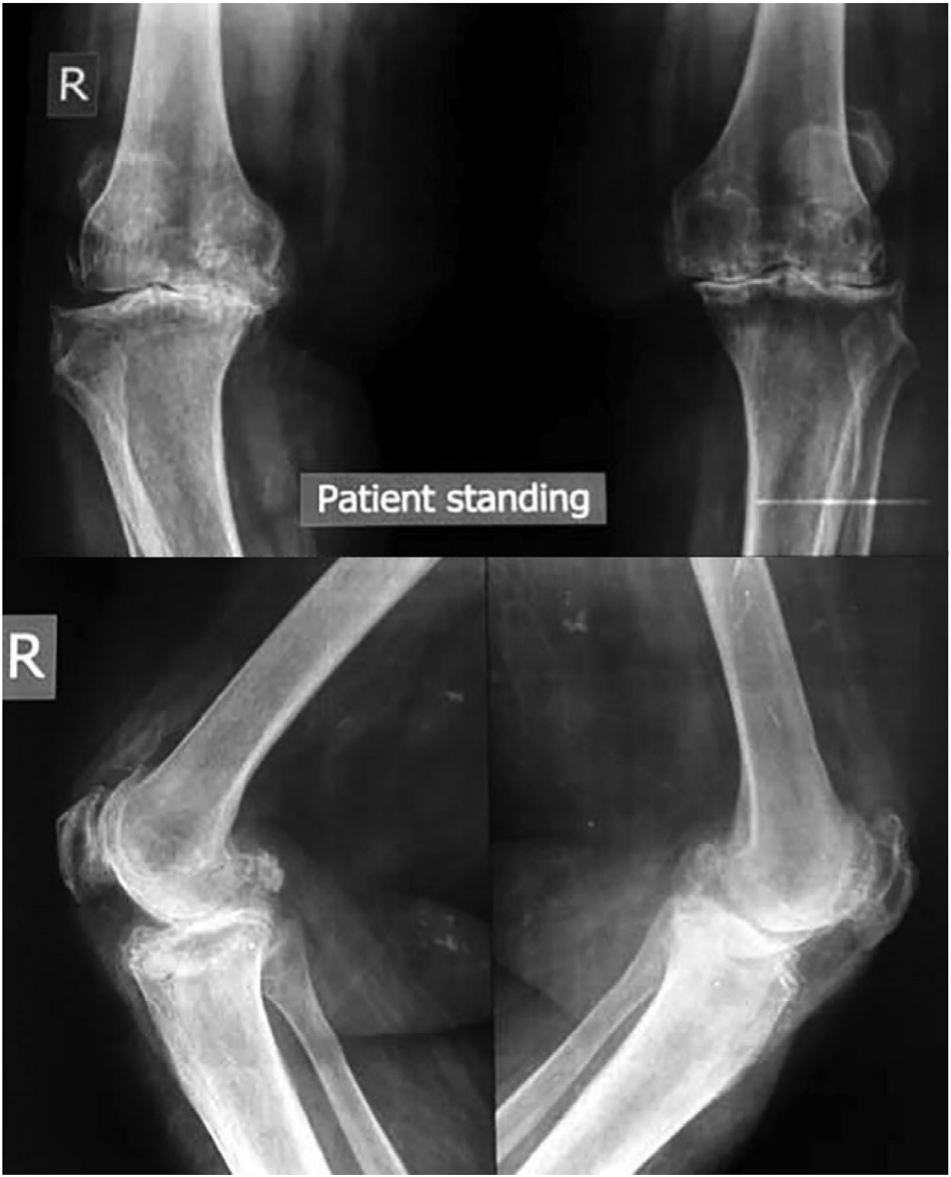
60 years old female, bilateral knee pain and difficulty walking. Preoperative x-rays showing advanced tricompartmental osteoarthritis and varus deformity.

**Fig. 3:**
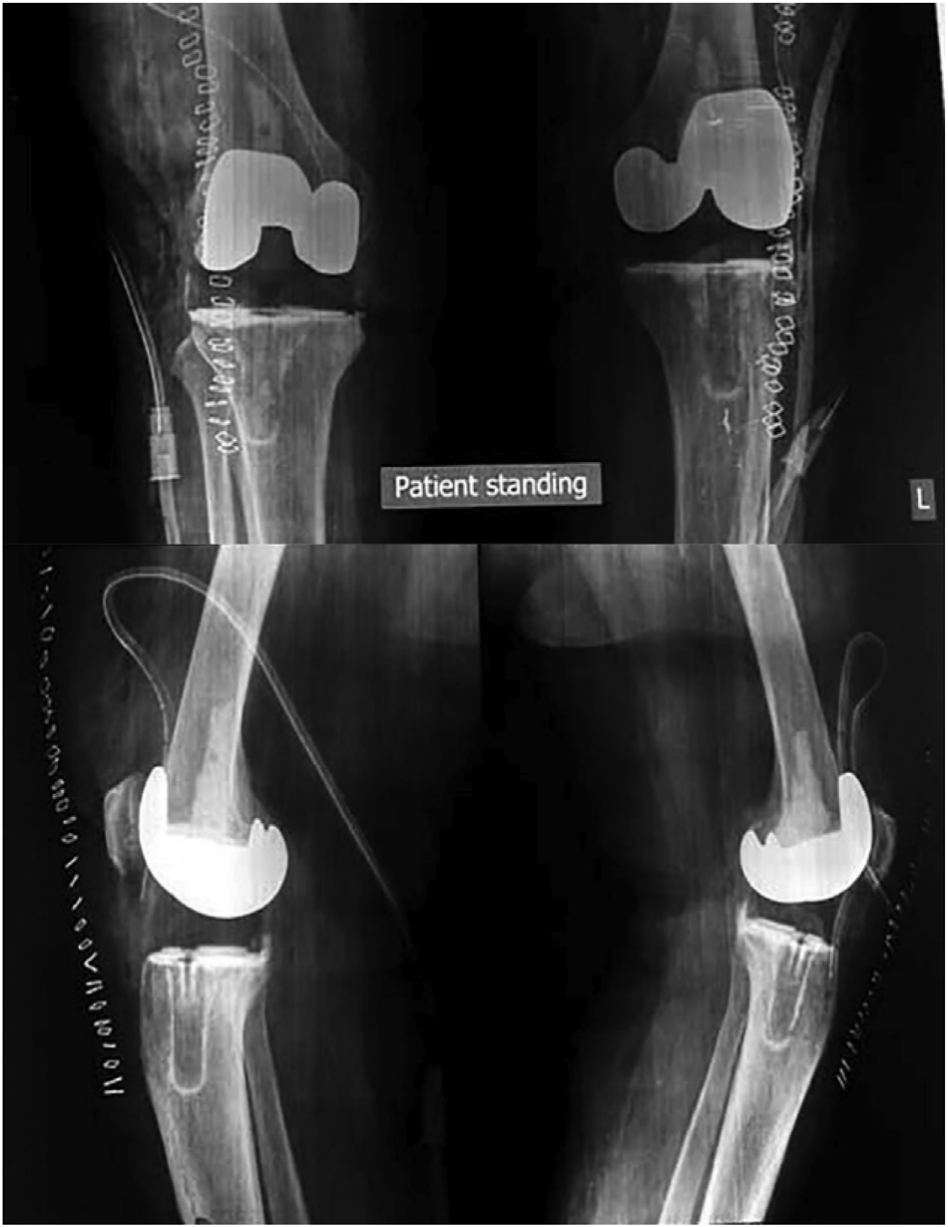
Postoperative x-rays of same patient showing the all poly tibia implant, restoration of joint space and coronal and sagittal alignment.

Considering the increasing prevalence of total knee replacement and financial crises especially in third world countries, orthopedic surgeons should utilize cost savings techniques without compromising the quality. Multiple randomized radio-stereo-metric, clinical outcome studies and two recent systematic reviews have suggested equivalence or superiority of the APT design over MBT in terms of failure [23]. In light of these results, it seems that the increased use of the APT design could save the healthcare system substantial amounts of money without compromising outcomes. In 2006 Muller and colleagues [24] proposed a possible cost savings of approximately 39 million dollars per year across England and wales, if just 50% of the 70,000 TKA performed annually used APTS. Fig. 2, Fig. 3 preoperative and postoperative x-rays showing a case of advanced tri-compartmental osteoarthritis where TKA done with APT design.

Authors also estimated that if all patients in their registry (16,500 total joints over a 14-year time period) aged ≥75 years had an APTC instead of a metal-backed tibia (MBT), the cost savings on implants alone would have amounted to $1.17 million [13].

Gioe and colleagues [13,25,26] have counted APTC's average cost less than the matching metFig. 2, Fig. 3al-backed component. James et al. [27] cited cost of primary knee replacement was on average $1000 less with APT Compared to MBT. Pomeroy et al. noted a 20%–30% cost difference concerning APTC and metal-backed tibia components [28]. Another important factor in overall cost effectiveness is the relative revision rate of the respective components. However, the best available data show that modern APTC have revision rates equivalent or superior to those of metal-backed implants. James et al. found the cost of revision was $21,650.34 and assumed to be the same regardless of the type of initial surgery [29].

### 2.3 Survivorship

Several authors have since documented excellent long-term success of the APTC in total condylar, posterior stabilized, and posterior cruciate condylar total knee prostheses [30,31]. Meta-analysis examined survival data from 16 published studies with around 6000 knees found no statistically significant difference in survival between APTC and metal-backed tibia groups [32]. Level I evidence comparing the APTC and metal-backed tibial has shown equivalent long-term outcomes [26,33,34]. No current prospective randomized study supports statistically significant survivorship outcomes between patients with metal-backed tibial components and APTC. Swedish knee arthroplasty registry (SKAR) has reported better results of APTC design over metal-backed tibial component in the PFC Sigma knee prosthesis about ten-year survival of the implant [29].

### 2.4 Backside wear and revision

A recent analysis of early retrievals reported no statistically significant difference in the visual appearance of backside damage between highly cross-linked and conventional liners [35]. SKAR have mentioned 416 of revisions that were in the metal-backed group out of 16,011 and 216 in the APTC group out of 11,722 [29].

### 2.5 Functions

Pomeroy et al. examined 298 APTC (average follow-up, 2.9 years) and mentioned no statically significant difference in clinical and functional scores between patients with APTC and cohorts with metal Backed tibial designs [28].

### 2.6 Infection

Polyethylene is known to support bacterial colonization and biofilm formation [36]. Therefore, removal of the insert may reduce bacterial load in the joint and theoretically improve the success rate of treatment in MBT. Acute deep infection of a TKA is commonly managed with surgical debridement and parenteral antibiotic therapy [37]. However, no clinical evidence at this time shows different infection eradication rates between APTC and metal-backed tibia components when debridement and component retention is undertaken.

### 2.7 Biomechanics

Polyethylene insert should be at least 8 mm in metal-backed tibial component to decrease surface wear [38,39]. Surgeon is bound to use smaller thickness polyethylene insert in metal backed as compared to isolated increased thickness polyethylene. In order to use large thickness insert with metal backed surgeon either has to do additional bone resection or to use smaller polyethylene [38,39].

### 2.8 Modularity of components

In terms of modularity Metal backed tibial (MBT) design does offers versatility of polyethylene insert that is advantageous particularly in younger patients, who might need revision surgery later on. But isolated polyethylene exchange have limited role in revision for addressing wear [40,41]^.^ In addition it can also address instability, requiring insert with additional constraint in revision surgery [42,43]. The MBT design provides different stem and augment alternatives that cannot be supplemented to the APTC, which are not utilized commonly in a primary TKA. In early acute hematogenous infection [44], liner exchange permits additional access to synovium, its additional removal and thus access to the implant interface but there is no interface in monoblock APTC. Tibial component can be removed more easily in APTC just by cutting the polyethylene, hence less chance of damaging the femoral component [45].

### 2.9 Patient selection

Candidates for APTC TKAs mainly low demand, such as the elderly (older than 70 years) or patients with rheumatoid arthritis [46,47]. Nonetheless, the APTC also has been recommended for younger patients [48,49]. Further studies are needed in this regard as by the year 2030, the expected number of patients younger than 65 years old who need to undergo TKA will reach 55% of total joint arthroplasty patients [56,57]. Regardless of age, APTC is as good as the MBT implant [58].

## 3 Conclusion

All polyethylene tibial component (APTC) is an appealing and cost effective alternative, and is associated with the excellent survivorship with low risk of revision. In light of the present-day economic evidence and long-term functional outcome, all-polyethylene can be a cost-effective alternative to the metal backed implant. Irrespective of age, APTC is as good as the MBT implant.

## Ethical approval

Review article applicable for exemption by our Ethical Review Committee ERC.

## Sources of funding

None.

## Author contribution

**Vickash Kumar:** design of study, first draft, methodology, final review and approval.

**Obada Hasan:** design, editing and writing of the manuscript, final review and approval.

**Masood Umer:** editing, overall supervision of the paper, final review and approval.

**Naveed Baloch:** editing with supervision and final approval of the writing.

## Funding and conflict of interest

Non-commercialized scientific review article. No funding from any source. All authors in this study declare no conflict of interest.

## Provenance and peer review

Not commissioned, externally peer reviewed.

## Trial registry number

Not applicable as this is a review article.

## Guarantor

All authors accept full responsibility for the work.

## Declaration of competing interest

No conflict of interest.
